# Robotic-Assisted XRF Testing System for In-Situ Areal Density Measurement of Light-Sensitive Explosive Coatings

**DOI:** 10.3390/s25123581

**Published:** 2025-06-06

**Authors:** Chang Xu, Haibin Xu, Ke Wu, Bo Chen, Pengju Dong, Yaguang Sui, Hai Chen

**Affiliations:** 1 National Key Laboratory of Intense Pulsed Radiation Simulation and Effect, Northwest Institute of Nuclear Technology, Xi’an 710034, China; 2 Department of Physics, Zhejiang University, Hangzhou 310027, China

**Keywords:** light-sensitive explosive, areal density, XRF, in situ measurement

## Abstract

The light-sensitive explosive (silver acetylide–silver nitrate, SASN) sprayed on structural surfaces can be synchronously initiated by intense pulsed flash, thereby simulating cold X-ray blow-off events characterized by thermal–mechanical coupling effects. By adjusting the areal density of SASN coatings, proportional blow-off impulse levels can be achieved. To address the challenge of in situ and non-destructive areal density measurement for SASN coatings, this study developed an X-ray fluorescence (XRF) detection system integrated with a six-axis spray robot. Excitation parameters (50 kV, 20 μA) and geometric configuration (6 cm focal distance) were optimized to establish a quadratic calibration model between Ag K_α_ counts and areal density (0–80 mg/cm^2^) with high correlation (R^2^ = 0.9987). Validation experiments were conducted on a uniformly coated SASN plate (20 × 20 cm) to evaluate the consistency between XRF and sampling methods. The XRF-measured areal density averaged 12.722 mg/cm^2^ with a coefficient of variation (CV) of 3.19%. The reference value obtained by the sampling method was 12.718 mg/cm^2^ (CV = 1.57%). The relative deviation between the two methods was only 0.03%, confirming the feasibility of XRF for the quantification of SASN coatings. The XRF system completed measurements in 1 h, achieving a 77.8% time reduction compared to conventional sampling (4.5 h), significantly enhancing efficiency. This work provides a reliable solution for in situ and non-destructive quality control of energetic material coatings.

## 1. Introduction

Energetic materials are substances that release energy through rapid chemical reactions, exhibiting wide applications in both civil and military fields [1,2,3,4]. Silver acetylide-silver nitrate (Ag_2_C_2_·AgNO_3_, SASN) is an energetic material, also known as a light-sensitive explosive [5,6,7,8]. Its stable storage in acetone facilitates the application of the SASN/acetone mixture onto the surface of a specific target via a spray robot [9]. Upon the evaporation of the acetone, a coating of SASN is formed on the target surface. Subsequently, this SASN coating can be instantaneously detonated by an intense light flash or pulse laser, which can be used to produce simulated cold X-ray blow-off impulse loading on a variety of targets to study the system’s structural response [10,11]. Pulse loading intensity directly correlates with the areal density of SASN.

The current measurement method of the areal density of SASN involves presetting sampling chips on the target surface, and determining the areal density by weighing the areal density of SASN on the sample chips before and after spraying [9]. However, traditional gravimetric methods exhibit limitations: acetone volatilization (requiring 4~5 h) delays measurement, while sampling chip placement disrupts coating distribution. The placement and removal of the sample chips will disrupt the distribution of SASN on the surface structure, and the measurement points are discrete, with the accuracy of the measurement depending on the number of preset points. Moreover, if the measurement result does not correspond to the designated areal density of SASN, the sample chips will need to be replaced on the target surface for respraying and subsequent re-measurement, a process that requires considerable time and effort. Therefore, it is essential to perform non-destructive, in situ, and real-time, precise measurement of the areal density of the SASN coating during the spraying process to ensure the accuracy of the loading procedure.

In recent years, with the increasing demand for non-destructive and in situ measurements, various testing technologies have been developed, such as XRF [12,13,14], infrared [15,16,17], ultrasonic [18,19,20,21], and laser [22,23,24,25]. One of the essential requirements for measuring the areal density of SASN coating is in situ and real-time, which can guide subsequent spraying based on the measured result to achieve the desired areal density distribution. During the spray process, the SASN coating is primarily composed of SASN and acetone. Due to the volatile nature of acetone, its content dynamically changes during the measurement process. Infrared testing technology mainly relies on analyzing temperature field changes on the structure surface after infrared radiation to ascertain thickness changes [15]. However, the continuous evaporation of acetone during the measurement can alter the temperature field, making real-time and accurate analysis impossible. Therefore, infrared testing technology is not suitable. Ultrasonic thickness measurement boasts strong penetration, portable equipment, and high measurement accuracy [18], but it requires the application of a liquid coupling agent on the coating surface, making it unsuitable for measuring the thickness of SASN. Laser thickness measurement involves scanning the coating surface with a laser beam and measuring the speed, brightness, and distribution characteristics of the reflected light generated after scanning to determine the coating thickness [22]. However, after integrating the laser thickness measurement system into the spray robot, the positioning accuracy of the robot may also have a certain impact on the measurement results.

X-ray fluorescence spectrometry (XRF) is a well-established technique for qualitative and quantitative analysis of materials, capable of analyzing all elements from sodium to uranium [26,27,28,29,30]. It is extensively utilized for elemental analysis, quantification of metallic components, and identification of post-detonation explosive residues [31,32,33]. Moreover, it can also achieve thickness or areal density measurement by emitting X-rays of a certain energy to the specimen and detecting the characteristic X-rays generated after the elements in the specimen are excited [12,13,14]. SASN, containing only silver as its metallic element, allows for the calibration of areal density by measuring the characteristic X-ray spectrum corresponding to its silver content. Furthermore, advancements in miniaturized X-ray light sources and high-precision detectors have resulted in X-ray equipment that is more compact, portable, and environmentally adaptable, making it easier to integrate with spray robots. Therefore, measuring the areal density of SASN using XRF is feasible.

In this study, we developed an XRF analytical system and integrated it with a spray robot. The robotic system uniformly applied synthesized SASN onto predefined sample chips to fabricate coatings with controlled areal densities (5–80 mg/cm^2^). Through systematic optimization of excitation parameters, including voltage (20–50 kV) and current (10–30 μA) based on Ag K_α_ intensity and signal-to-noise ratio (SNR) analysis, the optimal operational conditions were determined as 50 kV and 20 μA with 6 cm focal distance and 60 s sampling duration. A robust quadratic correlation was established between SASN areal density and Ag K_α_ counts. Validation results confirm that XRF provides high-precision measurements while substantially minimizing time expenditure. The robotic-integrated XRF system establishes a critical enabling technology for implementing non-destructive, in situ areal density characterization of SASN coatings in industrial settings.

## 2. XRF System

### 2.1. XRF Theory

During the spraying process, the SASN coating is composed of SASN and acetone, primarily containing elements such as Ag, C, H, O, and N. Due to the significantly higher atomic number and abundance of the Ag element compared to other elements, the intensity of characteristic X-rays generated by exciting Ag is much higher. Furthermore, the substrate material for SASN coating is Al (Z = 13), which has a significantly lower atomic number compared to Ag (Z = 47). Consequently, the characteristic X-ray intensity of Al is substantially weaker than that of Ag. Therefore, Ag is selected as the reference element. The excitation energies of the K and L shells of Ag are shown in Table 1. It is found that the excitation energy of the K shell of Ag is mainly in the range of 20 to 25 keV, while the excitation energy of the L shell is primarily around 3 keV. Moreover, the fluorescence yield of the K shell of Ag is much higher than that of the L shell, which means the K shell is more easily excited [34,35]. Furthermore, due to the higher probability of electrons transitioning from the L shell to the K shell compared to the M shell to the K shell, the intensity of the K_α_ peak in Ag elements is higher than that of the K_β_ peak. Therefore, the count of the K_α_ peak of Ag is used as the basis for evaluating the areal density of the coating.

### 2.2. XRF Testing System

**Figure 1:**
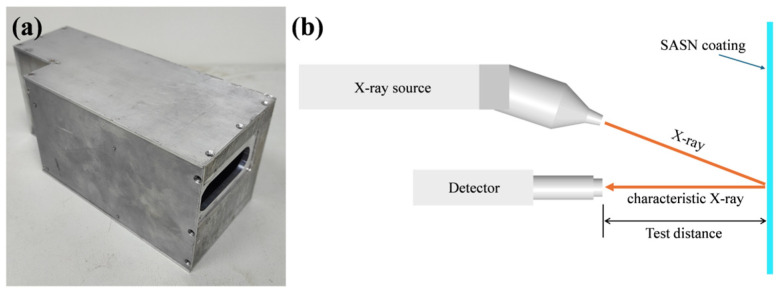
(**a**) XRF test device and (**b**) test system schematic diagram.

In order to achieve the in situ measurement during the spraying process. The testing system needs to be sufficiently compact to enable its integration into the spraying robot. Therefore, we have chosen a compact X-ray light source and detector, consisting of a Mini-X2 W target light source and X123 FAST SDD detector from the AMPTEK Company, respectively. Moreover, the X-ray light source is of the angled type, which is more conducive to controlling the size of the test system. The anode target material is W with a thickness of 1 μm, the Be window has a thickness of 125 μm, and the aluminum filter has a thickness of 254 μm. The specifications of the X-ray light source and detector are shown in Tables S1 and S2. In order to safeguard the test system, an aluminum protective cover with a test window was designed to prevent misoperations during the spraying process from damaging the testing system. Additionally, to avoid overheating of the XRF testing system due to prolonged operation, we have applied heat-conducting silicone grease at the points where the detector and radiation source come into contact with the protective cover, facilitating better heat dissipation. Considering that SASN is a highly sensitive explosive, to guarantee that the spraying system does not come into contact with the SASN coating during the measurement process, we designed a testing distance of 6 cm. Specifically, the distance from the focus of the X-ray source to the detector was set at 6 cm. The test system is shown in Figure 1. As shown in Figure 1b, the X-ray source generates high-energy X-rays that are directed towards the surface of the SASN coating. These X-rays interact with the atoms in the SASN coating, causing them to emit characteristic fluorescent X-rays, and the emitted X-rays are then detected by a suitable detector, which converts them into an electrical signal. The test distance is the distance between the detector and the SASN coating.

**Figure 2:**
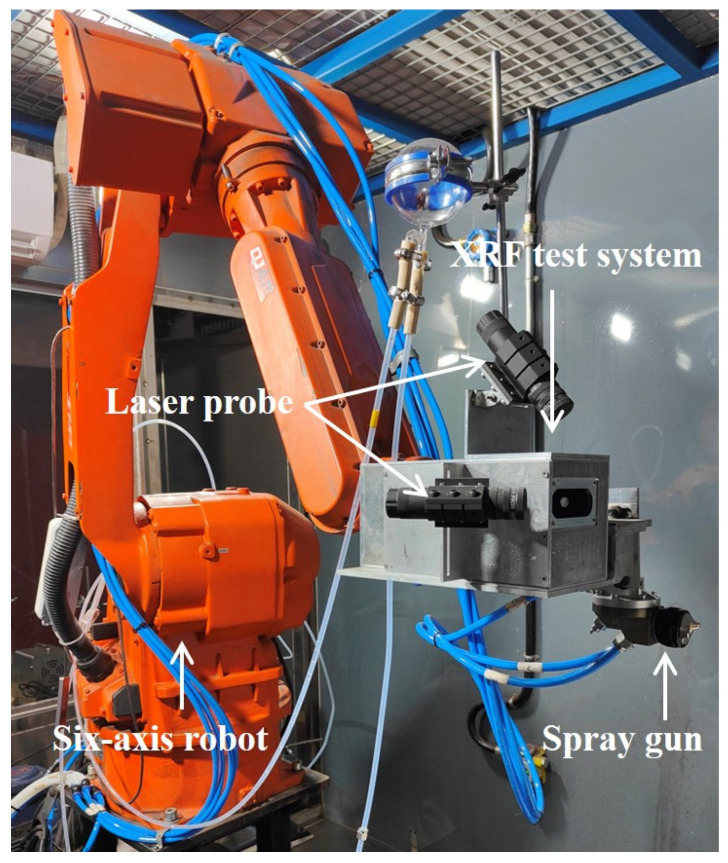
The spraying system.

Figure 2 shows that the XRF testing device is integrated into the spray robot. The spraying robot is the QJRB20-1 model from Qianjiang, China, and it is a six-axis robot. Detailed parameters of the spraying robot are provided in Table S3. Furthermore, it can be seen that there are two lasers positioned on the XRF testing system, with their focal points precisely aligned with the XRF measurement points. These lasers are primarily used to verify the accuracy of the planned measurement points of the robot during the testing process. During the spraying process, robots can be programmed to move to designated locations, where XRF measurements are then conducted to obtain the XRF spectra of SASN at those measurement points. Subsequently, the obtained areal density distribution calculated by the XRF spectra is used to guide the planning of spraying trajectories, ultimately achieving the designed areal density distribution.

## 3. Experimental and Analysis

### 3.1. SASN Production

The synthesis process of SASN is detailed in ref. [6]. Representative SEM micrographs, XRD patterns, and FT-IR spectra characterizing the SASN morphology and chemical composition are presented in Figure S1, with comprehensive spectral analysis provided in the Supporting Information.

### 3.2. SASN Coatings Production

**Figure 3:**
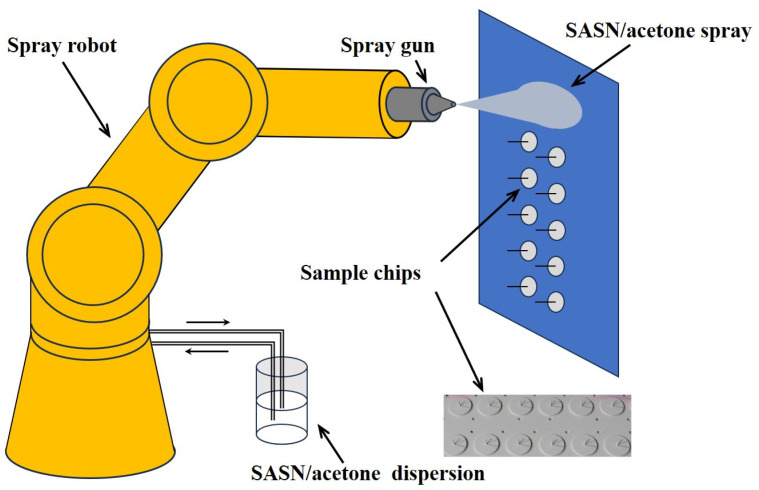
Schematic diagram for the preparation of SASN coatings.

As illustrated in Figure 3, a solution comprising 10 g of SASN dissolved in 500 mL of acetone is uniformly dispersed utilizing a homogenizer. Subsequently, the solution is sprayed onto the sample chips by manipulating the spray robot to follow a predetermined spray trajectory. The areal density of the SASN coating on the sample piece is regulated by adjusting the number of spray passes. Upon completion of the spraying process, the sample piece is meticulously removed, and any SASN adhering to the handle or side edges of the sample piece is eliminated. Following the application, the sample chips are taken out and placed in an oven at 55 °C for 10 h to eliminate the acetone, resulting in the formation of SASN coatings. The sample piece is made of aluminum, with a diameter of 2 cm and a thickness of 1 mm. It features a central handle that has a diameter of 1 mm and a height of 1 cm. A Kovar alloy sheet, measuring 2 cm in diameter and 0.1 mm in thickness, is adhered to the bottom surface to facilitate the magnetic attachment of the sample piece to the spray plate surface (specifically, the reverse side where magnets are installed). The spray process of the SASN coating is detailed in ref. [9].

### 3.3. XRF Data Collection

**Figure 4:**
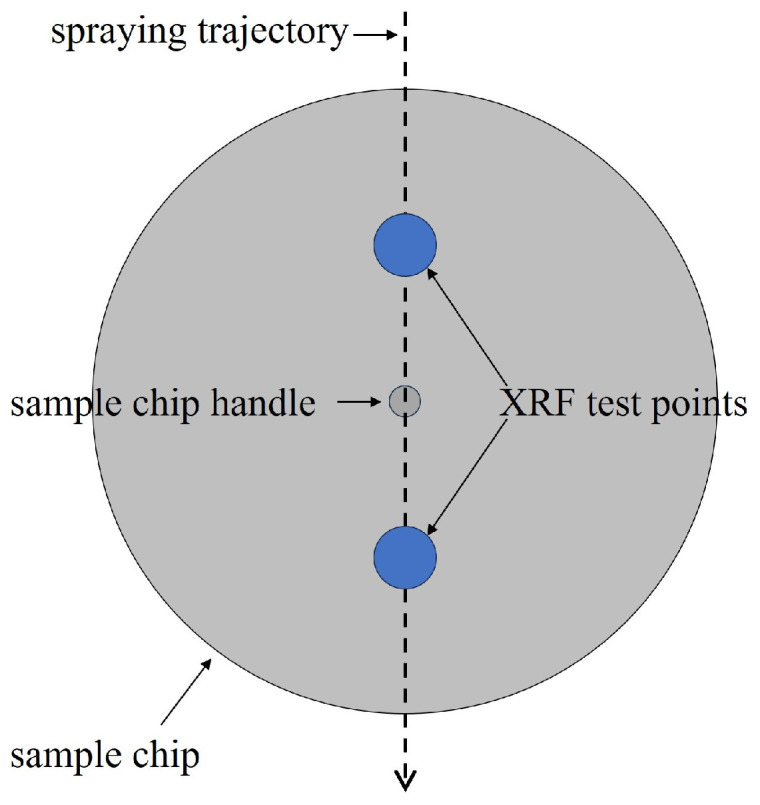
The schematic diagram of the XRF test points of the sample chip.

Considering the impact of the sample handle on the spray during the spraying process, the XRF measurement points were selected along the direction of the spraying trajectory, with one point taken above and one below the sample handle. A schematic diagram of the test points is shown in Figure 4. The sampling spot is a circle 2 mm in diameter.

**Figure 5:**
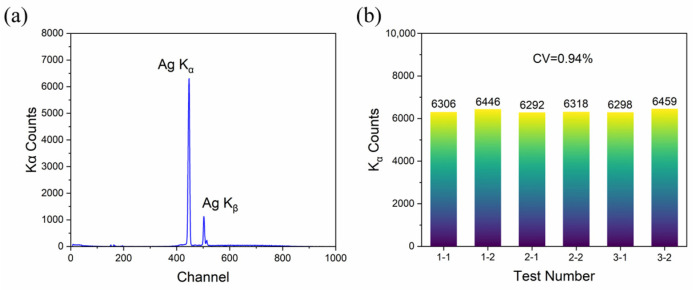
(**a**) XRF spectrum of the SASN coating (27 mg/cm^2^) under 50 kV, 20 μA, 6 cm, 60 s, and 6 cm conditions, and (**b**) the K_α_ peak counts of replicate experiments.

Figure 5a shows the XRF spectrum of the SASN coating with the areal density of 27 mg/cm^2^ under the following conditions: a test distance of 6 cm, 50 kV source voltage, 20 μA current, and 60 s sampling time. The characteristic Ag K_α_ and K_β_ peaks are clearly visible. To verify the stability of the XRF system, three replicate experiments with two test points shown in Figure 4, were conducted. The K_α_ peak counts from each experiment are shown in Figure 5b, demonstrating minimal variation between measurements. The calculated coefficient of variation (CV) for these data is approximately 0.94%, confirming the good stability of the test system.

**Figure 6:**
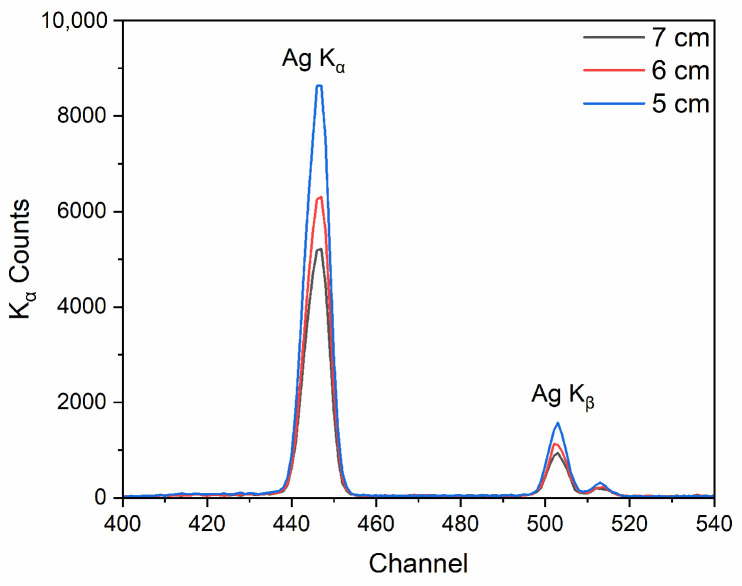
XRF spectrum of the SASN coating (27 mg/cm^2^) under 50 kV, 20 μA, 6 cm, and 60 s conditions with different test distances (5 cm, 6 cm, and 7 cm).

Figure 6 shows the XRF spectrum of the SASN coating with the areal density of 27 mg/cm^2^ under the conditions of different test distances (5 cm, 6 cm, and 7 cm), 50 kV source voltage, 20 μA current, and 60 s sampling time. The data reveal a gradual decrease in Ag K_α_ peak counts with increasing measurement distance. This phenomenon arises because the effective detection solid angle decreases quadratically with distance under the constraint of a fixed detector active area, leading to reduced X-ray photon flux. However, considering that SASN is an energetic material with high sensitivity, physical contact between the robotic probe and the SASN coating during testing may trigger accidental detonation. To mitigate this safety hazard, distances below 6 cm are not advisable. So the optimal testing distance was determined as 6 cm.

**Figure 7:**
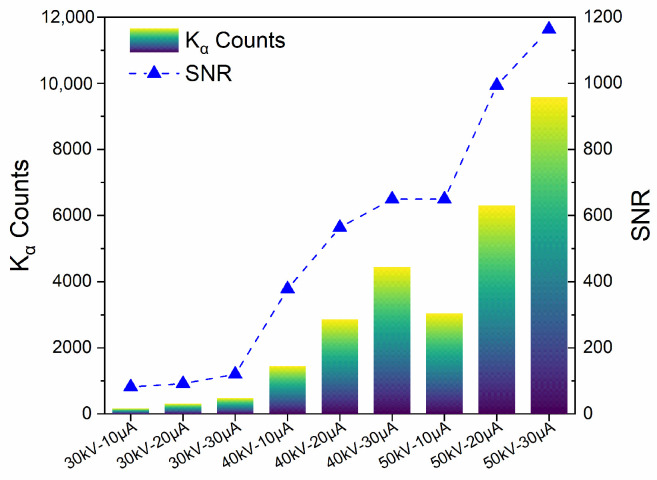
The K_α_ counts and SNR of different excitation voltages and currents.

The K_α_ peak counts and their signal-to-noise ratio (SNR) [36,37] are crucial factors for evaluating XRF measurements. Therefore, to ascertain the suitable excitation voltage and current, we conducted pertinent research on the same test sample by varying the voltage and current. The test condition and the corresponding XRF spectrum are shown in Table S4 and Figure S2. The K_α_ counts and SNR of different excitation voltages and currents are shown in Figure 7. It can be observed that, with the same voltage, as the current increases, the Kα counts gradually rise, indicating that increasing the current can more effectively excite the Ag element, resulting in the production of more K_α_ fluorescence photons. Furthermore, it can also be noted that the SNR improves with increasing current, further confirming that raising the current enhances the sensitivity and accuracy of the test. Meanwhile, when the current is constant, increasing the voltage has a similar effect. Additionally, power is another factor that must be considered during the test. Higher power results in a greater temperature rise during the operation of the XRF testing system, potentially impacting the accuracy of the XRF testing. Although the K_α_ counts and SNR peak under the condition of 50 kV and 30 μA, this entails higher energy consumption and a temperature increase. To alleviate the impact of temperature rise due to prolonged XRF operation, the optimal operating condition chosen is 50 kV and 20 μA, which consumes less energy while still maintaining high K_α_ counts and SNR.

### 3.4. Results and Discussion

**Figure 8:**
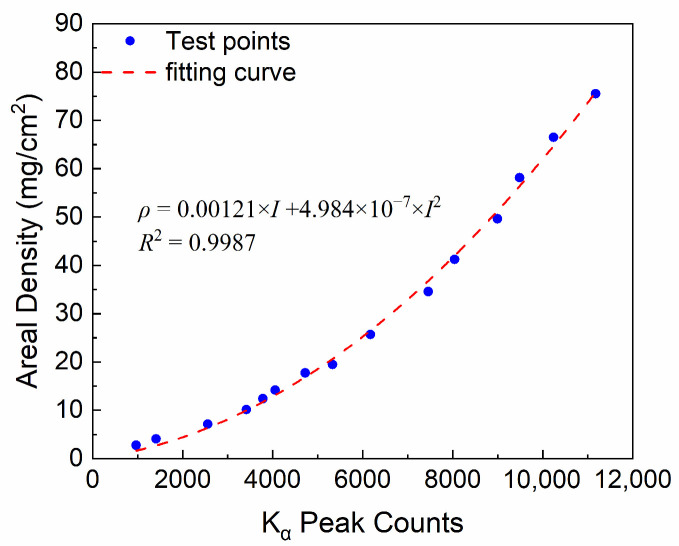
The test result and the fitting line between the Kα counts and the areal density.

Based on the above analysis, the final parameters selected for calibrating the SASN coatings with different areal densities are a voltage of 50 kV, a current of 20 μA, a test distance of 6 cm, and a sampling time of 60 s. We measured the XRF spectra of SASN coatings with different areal densities. The SASN coatings with different areal densities and the corresponding K_α_ counts are shown in Figure 8. It is found that an increase in areal density leads to a corresponding increase in K_α_ counts, due to the excitation of more silver ions by larger areal density. Moreover, the relationship between areal density (*ρ*) and K_α_ counts (*I*) is modeled by a quadratic function: *ρ* = 0.00121 × *I* + 4.984 × 10^−7^ × *I*^2^. And the correlation coefficient of 0.9987 demonstrates the excellent fit of the model. At low areal densities, a linear relationship exists between K_α_ counts and areal density. However, as areal density increases, this relationship transitions to a nonlinear one. In fact, a portion of the X-rays is absorbed by the SASN coating. As the areal density increases, so does the amount of absorbed X-rays. Consequently, it becomes evident that the rate of change in Kα counts decreases as areal density increases, indicating greater absorption of X-rays by the SASN coatings. This explains the saturation effect observed in the high areal density region, which aligns with the quadratic term in the fitting function. The fitted results will provide an effective means for non-destructive and in situ measurements of the areal density of the SASN coatings in the future.

**Figure 9:**
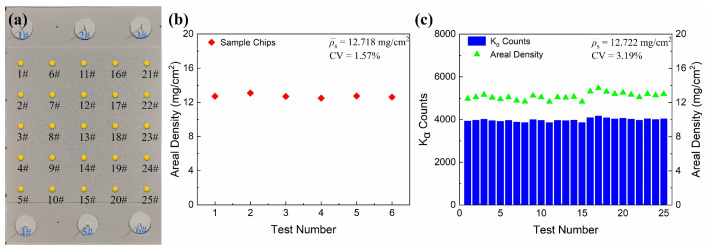
(**a**) Sprayed plates, sampling chips, and XRF measurement points, the areal density calculated by (**b**) sampling chips and (**c**) XRF measurements.

To validate the accuracy of the fitted curve, we conducted a uniformity spray experiment on a square plate with a side length of 20 cm, as shown in Figure 9a. On both the top and bottom surfaces of the plate, three sample chips were strategically placed to ascertain the areal density of the applied SASN coating after spraying. Additionally, 25 XRF measurement points were established on the plate, which were utilized to obtain XRF spectra from various locations. These spectra were subsequently analyzed to determine the areal density of the SASN coating at different measurement points. The XRF testing image is shown in Figure S3. Figure 9b,c show the test areal density calculated by sampling chips and XRF measurements. It can be observed that the average areal density of the SASN coating calculated based on XRF test data (12.722 mg/cm^2^) is largely consistent with the average areal density calculated from the sampled chips (12.718 mg/cm^2^), indicating that the XRF testing system is suitable for measuring the areal density of the SASN coatings. Furthermore, the coefficients of variation for the areal density measured by the sample chips and the XRF testing are 1.57% and 3.19%, respectively, indicating that the distribution uniformity of the areal density of the SASN coating sprayed on the flat plate is relatively good. Due to the larger number of measurement points in XRF testing, it can better reflect the areal density distribution of the SASN coating. This further validates that XRF testing can be used for non-destructive in situ testing of SASN coatings. Additionally, by predefining XRF measurement positions via the spray coating robot, the system completed 25-point measurements within 1 h, whereas the conventional sampling chips method required 4.5 h due to sequential processes of chips removal, drying, and measurement. This demonstrates that the robotic-assisted XRF-based approach achieves a 77.8% reduction in measurement time, substantially improving testing efficiency.

## 4. Conclusions

In summary, the SASN coatings with different areal densities were successfully fabricated by a spray robot. A customized XRF system was integrated into the spray robot to enable in situ measurement of the SASN coatings’ areal densities. By adjusting the parameters of the XRF testing system, distinct Ag K_α_ peaks could be observed in the XRF spectra of the SASN coatings. Repeatability tests with the same testing conditions demonstrated that the XRF testing system exhibits high stability, with a coefficient of variation (CV) of merely 0.94%. Through systematic analysis of Ag K_α_ peak counts, SNR, and power consumption under different testing conditions, the optimal parameters were determined as follows: voltage 50 kV, current 20 μA, testing distance 6 cm, and sampling time 60 s. And the relationship between the area density (0~80 mg/cm^2^) of SASN and the Ag K_α_ counts was obtained by testing different area densities of SASN. A robust quadratic calibration model was established: *ρ* = 0.00121 × *I* + 4.984 × 10^−7^ × *I*^2^ (*R*^2^ = 0.9987). Validation experiments were conducted on a uniformly sprayed SASN-coated plate (20 × 20 cm) using XRF (25 points) and sampling method (9 points). The results showed that the average areal density measured by XRF (12.722 mg/cm^2^) closely matched that from the sampling method (12.718 mg/cm^2^) with a relative error of 0.03%, confirming the accuracy of the calibration model. Furthermore, the XRF method significantly enhances testing efficiency, with a time reduction of 77.8% compared to the sampling method. This robotic-assisted XRF testing system provides a reliable way for in situ and non-destructive areal density measurement of the SASN coatings in industrial applications.

## Figures and Tables

**Table 1:** The excitation energy of Ag.

Element	K_α1_/keV	K_α2_/keV	K_β1_/keV	K_β2_/keV	L_α1_/keV	L_α2_/keV	L_β1_/keV	L_β2_/keV
Ag	22.163	21.990	24.942	25.456	2.984	2.895	3.150	3.347

## Data Availability

The original contributions presented in this study are included in the article/Supplementary Material.

## Supplementary Materials

The following supporting information can be downloaded at: https://www.mdpi.com/article/10.3390/s25123581/s1, Figure S1. (a) SEM, (b) size distribution, (c) XRD, and (d) FT-IR of SASN; Figure S2. The XRF spectrum with different test conditions; Figure S3. XRF testing image; Table S1. Technical specifications of X-ray light sources; Table S2. Technical specifications of the detector; Table S3. Technical specifications of the robot; Table S4. The test conditions.

## Abbreviations

The following abbreviations are used in this manuscript:

SASN	Silver acetylide–silver nitrate
