# Supplementary material for: Robotic-Assisted XRF Testing System for In-Situ Areal Density Measurement of Light-Sensitive Explosive Coatings
Source: Sensors (Basel). 2025 Jun 6;25(12):3581. doi: 10.3390/s25123581 (PMC12197155; doi:10.3390/s25123581)
Supplement: Supplementary file 1 [file sensors-25-03581-s001.zip › sensors-3654284-supplementary.pdf]

Robotic-Assisted XRF Testing System for In-situ Areal Density Measurement of Light Sensitive Explosive Coatings

Chang Xu[1], Haibin Xu[1,*], Ke Wu[1], Bo Chen[1], Pengju Dong[1], Yaguang Sui[1] and Hai Chen[2]

[1] National Key Laboratory of Intense Pulsed Radiation Simulation and Effect, Northwest Institute of Nuclear Technology, Xi'an 710034, China;

[2] Department of Physics, Zhejiang University, Hangzhou 310027, China

[*] Correspondence: xuhaibin@nint.ac.cn

Table S1. Technical specifications of X-ray light sources

**Technical index**	**X-ray source**
Rated power	10W
Maximum voltage	50kV
Maximum current	200μA
Total weight	400g
Collimator aperture	2mm
Filter material	Al
Filter thickness Al (μm)	254
Filter thickness Be (μm)	125
Operating temperature	-10~50℃

Table S2. Technical specifications of the detector

**Technical index**	**Detector**
Rated power	2W
Maximum voltage	220V
Maximum current	25μA
Total weight	180g
Resolution	5.9 keV，123 eV
Detector material	Silicon drift detector
Detector size (mm^2)	70 (Circle)
Thickness (μm)	500
Operating temperature	-35~80℃

Table S3. Technical specifications of robot

Mechanism	Vertical multi-joint robot
Axis number	6
Payload	20 kg
Max armspan	1671 mm
Repeat positioning	±0.05 mm
Protection degree	IP67, ExdpxIIBT4Gb

Motion range	J1 ±172°	
	J2	±158°, -110°
	J3	±83°, -92°
	J4	±170°
	J5	±125°
	J6	±360°
Max speed	J1	150°/s
	J2	150°/s
	J3	150°/s
	J4	281°/s
	J5	292°/s
	J6	372°/s
Allowable torque	J4	38.81 N·s
	J5	38.81 N·s
	J6	29.4 N·s
Inertia Moment	J4	3 kg/m^2
	J5	3 kg/m^2
	J6	2.2 kg/m^2

The SEM of SASN is shown in Fig. S1(a), it can be found that the micromorphology of SASN consists of nanospherical particles and the particle size distribution of SASN is relatively uniform. According to Fig. S1(b), the particle size distribution range is approximately between 400 nm and 600 nm. Fig. S1(c) presents the XRD spectrum of SASN, it can be observed that the positions and intensities of the characteristic peaks of SASN are in perfect agreement with the standard crystal structure (PDF#03-0692), confirming the successful synthesis of SASN. Fig. S1(d) exhibits the FT-IR spectrum of SASN, it can be found that peaks at 1303 cm^{-1} and 802 cm^{-1} wavenumbers of SASN are assigned to the antisymmetric stretching vibration and bending vibration modes of the nitrate ion (NO_3^-), respectively. However, it can be seen there are very weak absorption peaks of the acetylene bond (−C≡C−) in range of 2500-2000 cm^{-1}, which are attribute to the non-polar nature of the acetylene bonds and the highly symmetrical

molecular structure of SASN.

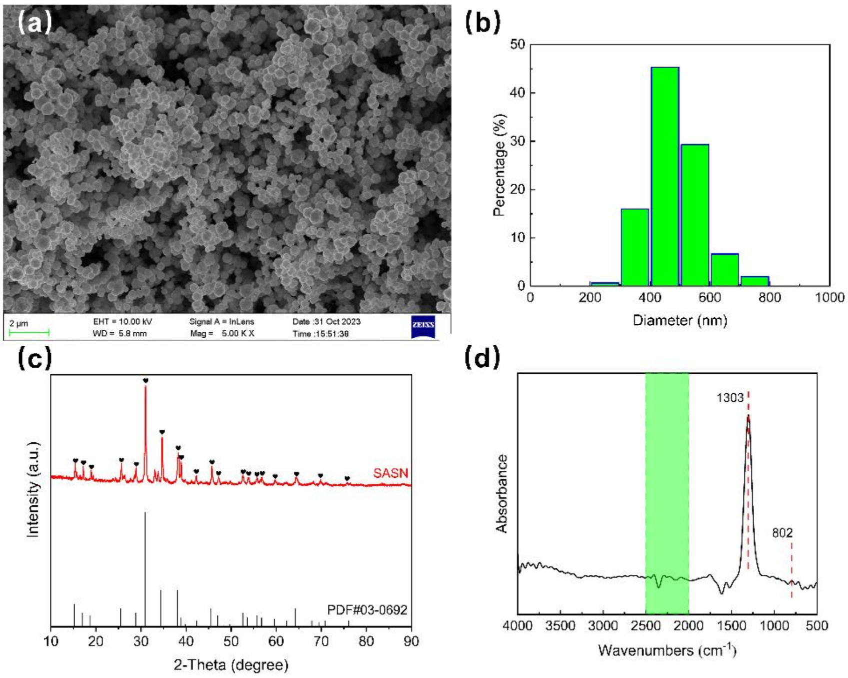

Figure S1 (a): SEM, (b): size distribution, (c): XRD and (d): FT-IR of SASN

Table S4. The test condition

No.	Areal density (mg/cm^2)	Test distance (cm)	Voltage (kV)	Current (μA)	K_α Counts	SNR
1		6	30	10	166	81.56
2		6	30	20	323	91.45
3		6	30	30	481	119.96
4		6	40	10	1451	377.93
5	27	6	40	20	2856	564.07
6		6	40	30	4447	649.31
7		6	50	10	3045	649.53
8		6	50	20	6306	993.24
9		6	50	30	9585	1163.16

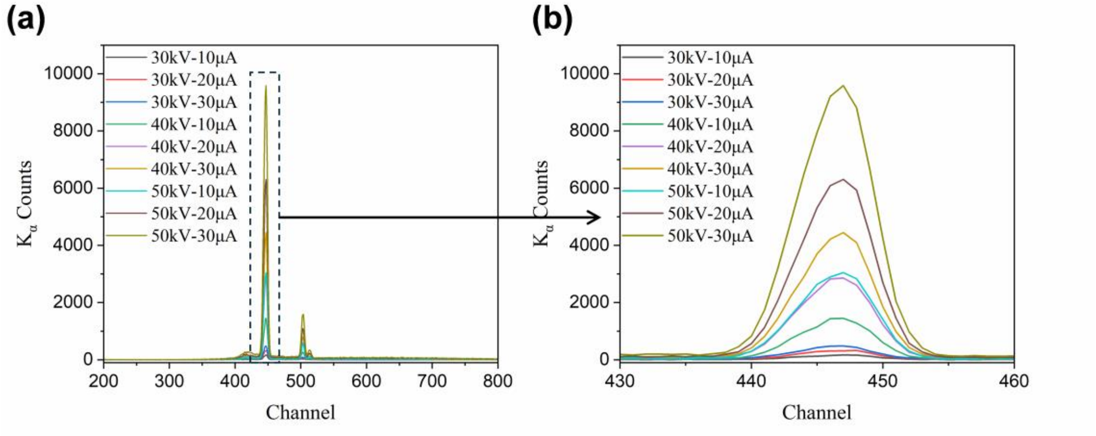

Figure S2 The XRF spectrum with different test condition

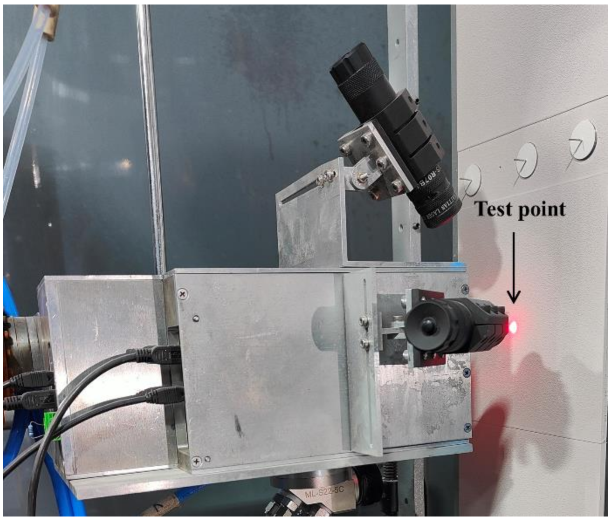

Figure S3 XRF testing image
